# Characterization of the Native Oxide Shell of Copper Metal Powder Spherical Particles

**DOI:** 10.3390/ma15207236

**Published:** 2022-10-17

**Authors:** Morsi M. Mahmoud

**Affiliations:** 1 Mechanical Engineering Department, College of Engineering, King Fahd University of Petroleum and Minerals, Dhahran 31261, Saudi Arabia; morsimahmoud@kfupm.edu.sa; 2 Interdisciplinary Research Center for Advanced Materials, King Fahd University of Petroleum & Minerals, Dhahran 31261, Saudi Arabia

**Keywords:** Cu oxide native layer, thickness, inhomogeneous, AES, dual-beam FIB-SEM

## Abstract

The native oxide layer that forms on copper (Cu) metal spherical particle surfaces under ambient handling conditions has been shown to have a significant effect on sintering behavior during microwave heating in a previous study, where an abnormal expansion was observed and characterized during sintering of Cu compacts using reducing gases. Because microwave (MW) heating is selective and depends greatly on the dielectric properties of the materials, this thin oxide layer will absorb MW energy easily and can consequently be heated drastically starting from room temperature until the reduction process occurs. In the current study, this oxide ceramic layer was qualitatively and quantitatively characterized using the carrier gas hot extraction (CGHE) method, Auger electron spectroscopy (AES), and a dual-beam focused ion beam (FIB)/scanning electron microscope (SEM) system that combines both FIB and SEM in one single instrument. Two different commercial gas-atomized spherical Cu metal powders with different particle sizes were investigated, where the average oxygen content of the powders was found to be around 0.575 wt% using the CGHE technique. Furthermore, AES spectra along with depth profile measurements were used to qualitatively characterize this oxide layer, with only a rough quantitative thickness approximation due to method limitations and the electron beam reduction effect. For the dual-beam FIB-SEM system, a platinum (Pt) coating was first deposited on the Cu particle surfaces prior to any characterization in order to protect and to preserve the oxide layer from any possible beam-induced reduction. Subsequently, the Pt-coated Cu particles were then cross-sectioned in the middle in situ using an FIB beam, where SEM micrographs of the resulted fresh sections were characterized at a 36° angle stage tilt with four different detector modes. Quantitative thickness characterization of this native oxide layer was successfully achieved using the adapted dual-beam FIB-SEM setup with more accuracy. Overall, the native Cu oxide layer was found to be inhomogeneous over the particles, and its thickness was strongly dependent on particle size. The thickness ranged from around 22–67 nm for Cu powder with a 10 µm average particle size (APS) and around 850–1050 nm for one with less than 149 µm.

## 1. Introduction

Processing materials using microwave (MW) energy can offer a wide range of advantages [1,2,3,4] if it is properly implemented, as compared to other processing techniques. In fact, it had been successfully used to process many materials [5,6,7,8,9,10,11,12,13,14,15,16,17,18] in different fields recently, such as but not limited to coal, oxide and carbide ceramics, composites, cement and concrete, alloys, fly ash, and even in CO_2_ adsorption. Furthermore, MW energy has been used to heat and to sinter metals and their alloys [19,20,21,22,23,24,25,26], from the early reported works where MW was used to heat metals to the most recent reported studies where it has been used to join metals and alloys, but its full use needs a comprehensive understanding of MW–material interactions.

The MW absorption of a given material depends significantly on how much as well as how fast the MW energy can be absorbed within a given material. The rate of MW energy absorption can be expressed in terms of power per unit volume [1], as shown in the following equation:

P_a_ = ω ε_o_ ε″_eff_ E^2^_rms_ + ω µ_o_ µ″_eff_ H^2^_rms_ (watts/m^3^) (1)

where the term (ω ε_o_ ε″_eff_ E^2^_rms_ ) is related to electric losses and the term (ω µ_o_ µ″_eff_ H^2^_rms_) is related to magnetic losses. The other terms are defined as follows: ω is the angular frequency, ε_o_ is the permittivity of free space, ε″_eff_ is the effective relative dielectric loss, E_rms_ is the root mean square of the internal electric field, µ_o_ is the permeability of free space, µ″_eff_ is the effective relative magnetic loss, and H_rms_ is the root mean square of the magnetic field strength.

The effective relative dielectric loss can be expressed as shown below in Equation (2).

ε″_eff_ = ε″_c_ + ε″_s_ + ε″_d_ + ε″_I_ + ε″_e_ (2)

where ε″_c_ is the loss due to DC conductivity and the last four terms represent dielectric losses due to polarization mechanisms, where ε″_s_ is for space charge (interfacial); ε″_d_ is for dipolar; ε″_I_ is for ionic, and ε″_e_ is for electronic components.

It was experimentally demonstrated that the thermodynamically stabilized native thin oxide layer that forms at normal ambient handling conditions on copper (Cu) metal particle surfaces has a vital role in the sintering behavior, microstructural changes, and MW–material interaction of that metal powder during high-frequency MW processing [27]. These experimental observations and findings could explain why metal powders can be heated using MW energy while bulk metals reflect MWs and cannot heat at room temperature. The MW absorption and behavior of metal powders is different from their bulk metals at room temperature [19,28]. This thin oxide layer is considered a dielectric ceramic layer [29] that covers an electrically conductive metallic particle, so those particles are actually similar to a composite material structure. Several studies have investigated microwave heating of metal powders [26,30,31,32,33,34,35,36,37,38,39]. Furthermore, several recent theoretical studies were performed using effective-medium approximation models, where MW heating of electrically conductive powder particles surrounded by insulating oxide layer was investigated [33,36,40]. Identifying the interaction of MW energy with a given electrically conductive metal particle with an insulated dielectric thin ceramic oxide layer is vital for a better understanding of many material processing processes and extremely needed for an accurate modeling of metal powders or metal-matrix composites. The effective dielectric and effective magnetic properties are key in the numerical modeling of MW processing of materials, and for any other similar processes that involve heating of metal particle using electromagnetic waves.

In the previously reported work [27], it was experimentally shown that an abnormal expansion of Cu metal compacts occurred during the early stages of an MW sintering process of Cu metal particles under hydrogen (H_2_) atmosphere where an in situ MW dilatometry measurement setup was used. The observed expansion was due to the formation and effect of a superheated water vapor that was formed as a byproduct of the reduction process of the native ceramic thin oxide layer by the H_2_ gas atmosphere. Furthermore, that vapor formation caused the formation of cracks in the Cu particles during the early stage of microwave sintering before they were completely healed up again at later higher-temperature heating stages. Preliminary characterization of the native thin oxide layer was performed using X-ray photoelectron spectroscopy (XPS) that revealed the existence of two types of Cu oxides: Cu^+2^ and Cu^+1^. The thickness of the thin oxide layer was roughly estimated using XPS depth profiling, as an accurate thin layer thickness characterization could not be estimated due to the beam-induced reduction effect of the XPS depth-profiling process and also due to XPS process limitations [41].

Several methods have been used to study different thin films in different fields. For example, small-angle X-ray scattering (SAXS) was used as a non-invasive characterization tool for nanostructured and functionalized particles [42]. Furthermore, several copper oxide films were studied using Kelvin probe force microscopy (KPFM) and conductive AFM (C-AFM) [43], where different resistivity values were reported for the different oxides studied. Other studies used high-resolution transmission electron microscopy (HRTEM) to study the effect of non-uniform Cu oxide layers, around several hundred nm thick, on the surface of 10 μm-diameter Cu/SnAg microbumps [44]. In addition, controlled CuO films produced via a controlled diode laser technique were characterized using different material characterization techniques, including TEM [45].

The goal of this work was to experimentally, qualitatively, and quantitatively characterize, with more accuracy, the native thin ceramic oxide layer that thermodynamically forms on Cu metal surfaces for two differently sized spherical particles. It is believed that this study will help to enhance the fundamental understanding of the MW–material interaction during metal powder sintering. Furthermore, it will be very useful in numerical models to accurately estimate the effective dielectric and magnetic properties of a given metal powder during electromagnetic heating, for better control of microwave processing in metals sintering. Moreover, it can be used in other related fields where Cu metal powders or any other similar metal particles are being heated under any kind of electromagnetic radiation.

## 2. Material and Experimental Work

### 2.1. Cu Metal Powder Specifications

Two commercial gas-atomized spherical Cu metal powders with different particle sizes from Alfa Aesar were used in the current study; particles with a size less than 149 µm and particles with an average particle size (APS) of 10 µm were investigated. More detailed specifications of the two Cu metallic powders used are given in Table 1.

### 2.2. Characterizations of As-Received Cu Powder

The as-received Cu powders were characterized using X-ray diffraction (XRD) and scanning electron microscopy (SEM). X-ray diffraction was performed using a Seifert C3000 powder diffractometer (CuKa radiation) while scanning electron microscopy (SEM) was conducted using a Hitachi S800 and a Philips XL40, which were equipped with energy-dispersive X-ray spectroscopy (EDX) systems.

Furthermore, the oxygen content of the as-received Cu powders was chemically analyzed with the carrier gas hot extraction (CGHE) method, using a TC600 (LECO) commercial oxygen/nitrogen analyzer. The analyzer was first calibrated with the certified standard JK 47, a steel powder from Sweden. The calibration was then verified with a copper standard (91000-1002) from ELTRA. The calibration range was a close match with the standard sample concentration used. The standards and the samples were weighed, with a mass ranging from 5 to 30 mg with a weighing accuracy of ±0.002 mg, then placed into a high-temperature graphite crucible between two electrodes for outgassing using 5800 watts (W) where the measurements took place. The evolving CO_2_ and CO gases were then swept out by helium (He) as an inert gas carrier and measured via non-dispersive infrared photometry (NDIR).

### 2.3. Auger Electron Spectroscopy

Characterization of the native thin oxide layer on Cu metal powder particle surfaces was performed using Auger electron spectroscopy. For AES measurements, Cu spherical particles with a size of less than 149 µm were pressed into an indium (In) foil. The electron beam analysis was done under the following conditions: vacuum of ultra-high voltage (UHV) of 3 × 10^−10^ Torr, accelerating voltage of 10 keV, current of 20 nA, beam size of 24 nm, and sample tilting angle of 0° from surface normal to electron gun. Furthermore, depth profiles on Cu particles were performed via argon (Ar) ion beam for sputtering/etching, where the reported depths are based on sputter rates for the SiO_2_ standard, while the real etch rates are heavily dependent on the characteristics of each material. The following analysis conditions were adapted as follows: UHV at sputtering of 3 × 10^−9^ Torr, accelerating voltage of 250 eV, Ar ion current of 500 nA, etching area of 1 × 1 mm, sample tilting angle of 15° from surface normal to ion gun, and etching rate of 0.5 nm/min for SiO_2_.

### 2.4. Dual-Beam SEM-FIB Characterization

**Figure 1:**
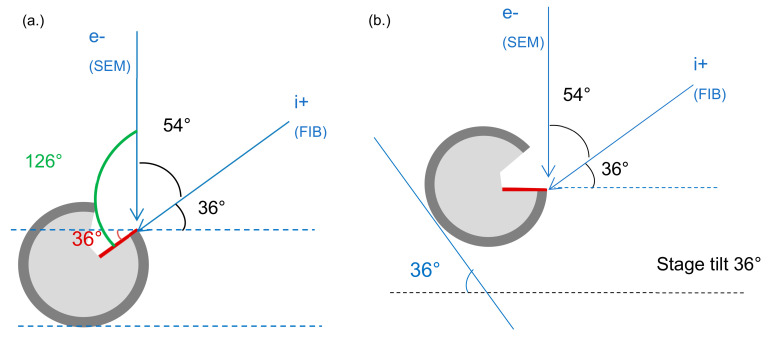
Schematic geometry of (**a**) Dual beam FIB-SEM configuration with particle cross-sectioning process using FIB at 0° angle stage tilt and (**b**) Cross-section observation at 36° angle stage tilt using SEM setup.

The as-received Cu metal particles were also characterized using a dual-beam FIB-SEM system, a Zeiss Auriga 60 DualBeam FIB. The system is a combination of a scanning electron microscope (SEM) and a focused ion beam (FIB) unit that allows imaging and structure characterization at a nanoscale level in several materials. A focused gallium (Ga) ion beam was used for ion imaging and to slice any predefined sections of the investigated Cu particles. At the same time, the SEM was used to image the oxide layer structure that was revealed by the FIB. To avoid any beam-induced reduction and for more accurate oxide layer thickness estimation, Cu particles were first coated with a Pt layer from precursor gases using the electron or the ion beam prior to any thin oxide layer thickness measurements. Pt-coated particles were then cross-sectioned in the middle using the FIB at a 0° stage tilt, and the resulting fresh sections were then observed via the SEM with a stage tilt of 36°. The resulting cross-section surfaces were perpendicular to the SEM beam so that no tilt correction was needed, as shown and schematically explained in Figure 1. The operating voltage condition for the dual system were high-tension SEM: 200 V–30 kV and high-tension FIB: 0.2 kV–30 kV, with an electron beam resolution of 1 nm at 15 and 30 kV. Four different detectors were used during FIB-SEM measurement. A secondary electron and secondary ion detector (SESI–Everhart-Thornley type) was used for secondary electron images; an InLens (immersion lens) detector was used for high-efficiency secondary electron (SE) images, where a voltage bias was applied, allowing for backscatter electron (BSE) images as well as SE images or mixed types; a four-quadrant solid state backscatter detector (NTS BSD) was used for backscatter electron images; and an energy-selective backscatter (ESB) detector was used for backscatter electron images.

## 3. Results and Discussions

**Figure 2:**
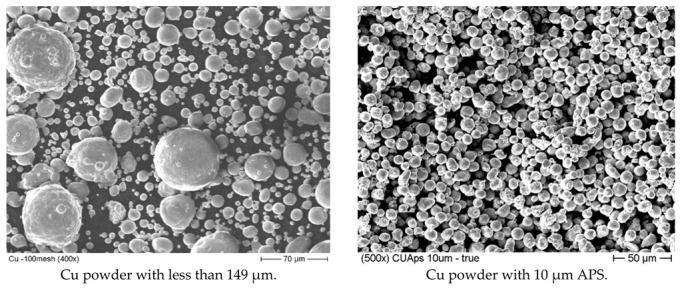
SEM micrographs of the two different particle sizes of as-received spherical Cu powders.

**Figure 3:**
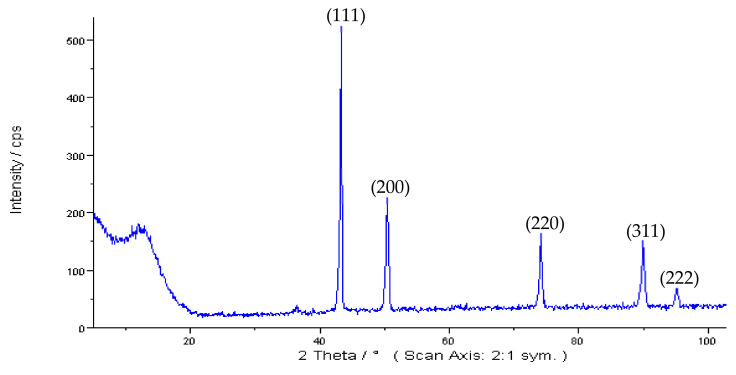
XRD micrograph of the spherical Cu powders.

Figure 2 shows SEM micrographs of the as-received spherical Cu powders with 10 µm APS and with less than 149 µm particle sizes. As shown in the graphs, both powder types had the typical gas-atomized spherical shape. Figure 3 shows the measured indexed XRD pattern of the as-received Cu powders. Both types of investigated powders showed similar patterns, where the resulting XRD peaks of the powders match very well with the typical face-centered cubic (FCC) crystal structure of copper metal with a lattice parameter of 3.61500 Å and a Fm-3m (No. 225) space group [46]. The XRD pattern showed no detection or significant presence of any type of Cu oxide peaks, which can be explained and attributed to the detection limits of the XRD technique [47].

Table 2 shows the oxygen content measurements of the two different Cu powders using the carrier gas hot extraction (CGHE) method, with a detection limit of 0.006 wt%. The mean values of several measurements for the two investigated Cu powder samples were found to be 0.573 wt% for the particles with 10 µm APS and 0.578 wt% for the ones with less than 149 µm. Those oxygen content mean values were somewhat close to the earlier reported values measured via TGA analysis in a previous study [27] where it was indicated that 0.49% weight loss had occurred. That minor difference could be attributed to the accuracy and the detection limits of both methods used. In general, based on both methods, the oxygen content of the Cu powder particles is in the range between 0.5–0.6 wt%.

**Figure 4:**
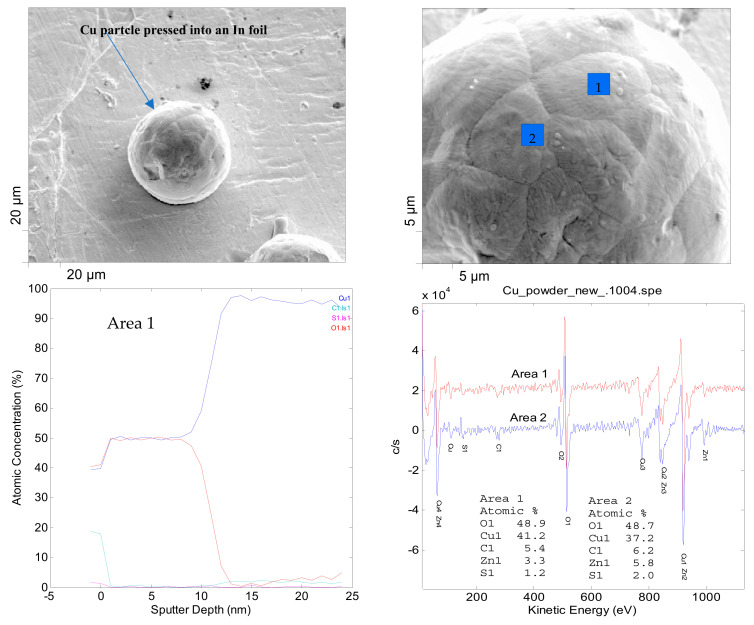
AES measurements of Cu particles with less than 149 µm.

Figure 4 shows AES measurements with depth profiling of Cu particles with less than 149 µm particle size. The depth profiling was done on a few selected particles. The sputter depth was around 11–13 nm for the spot marked “area 1” on the specific particle shown in Figure 4. It was observed that the depth profile varied from one spot to another within the same particle (areas 1 and 2). This implies that the oxide layer distribution is inhomogeneous throughout the same particle. The kinetic energies of the emitted AES electrons are also shown in Figure 4, which are characteristic of different types of elements present within the top few nanometers (3–10 nm) of the particle surface. The Cu oxide thin layer was qualitatively identified in AES measurements in addition to some minor impurities due to contamination. Furthermore, AES measurement and depth information are subject to certain limitations due to the fact that AES electrons can escape only from certain limited depths (typically 1–5 atomic monolayers, ~3–10 nm depth) and this requires calibration of both the measured signal intensities and the sputtering timescale [41,48]. Therefore, the oxide layer thickness can not be quantitatively estimated accurately using AES since it is a surface-sensitive technique. Still, it provides a reliable qualitative and semi-quantitative characterization of the thin oxide layer.

**Figure 5:**
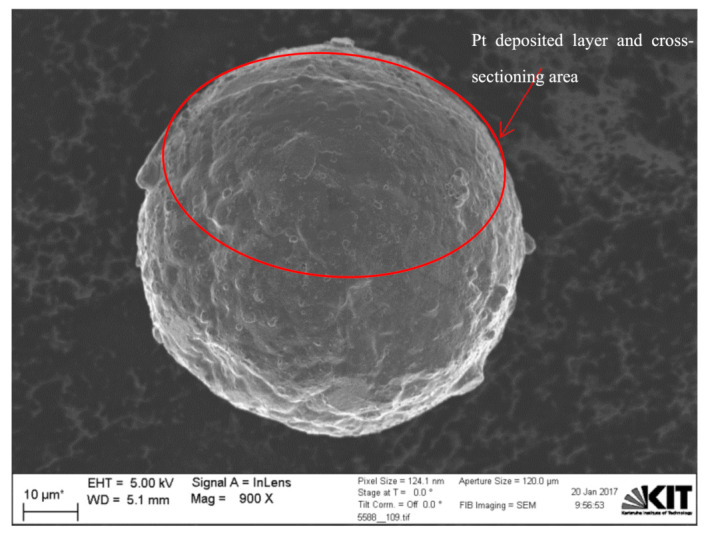
Pt coating and cross-sectioning area of a Cu particle using dual beam FIB-SEM characterization.

Figure 5 shows the deposited platinum (Pt) layer and a schematic of the cross-sectioning process using the FIB beam during the dual-beam FIB-SEM system characterization of Cu powder particles. The Pt layer was first deposited on the particle prior to any thin oxide layer characterization to protect that layer from any electron or ion beam-induced reduction [49]. Furthermore, FIB sectioning was performed with a 0° angle stage tilt, while SEM observation was done later with a 36° angle stage tilt, so no tilt correction was needed to measure the oxide layer thickness, as shown in Figure 1.

**Figure 6:**
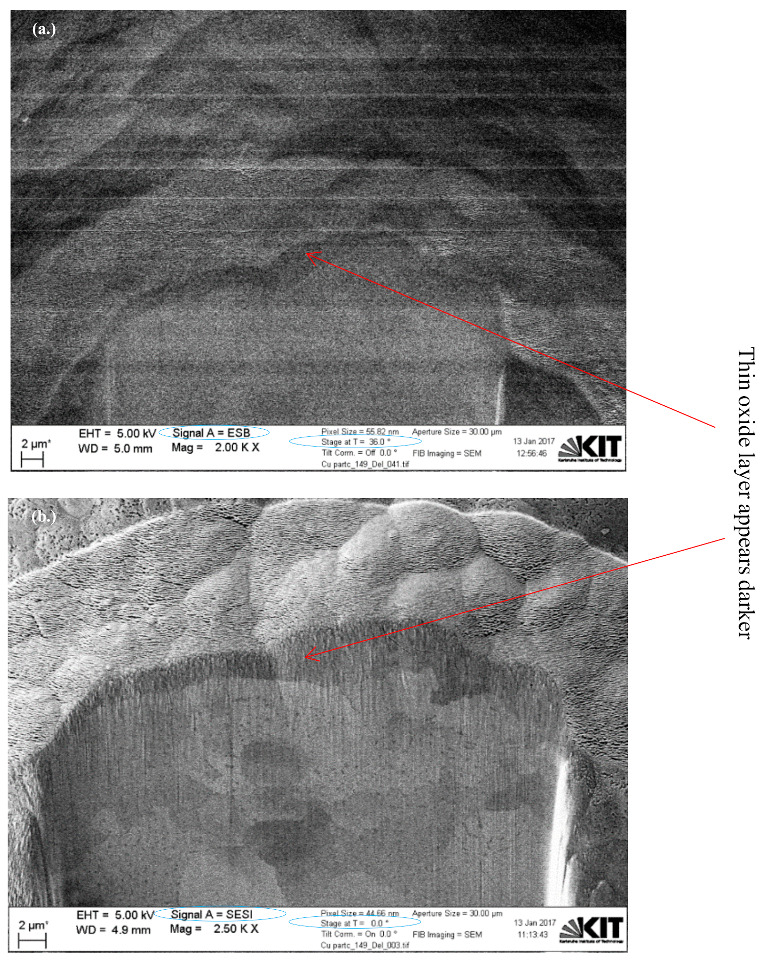
Dual FIB-SEM sectioned images of Cu particles with less than 149 µm using (**a**) BSE and (**b**) SESI modes for comparison.

**Figure 7:**
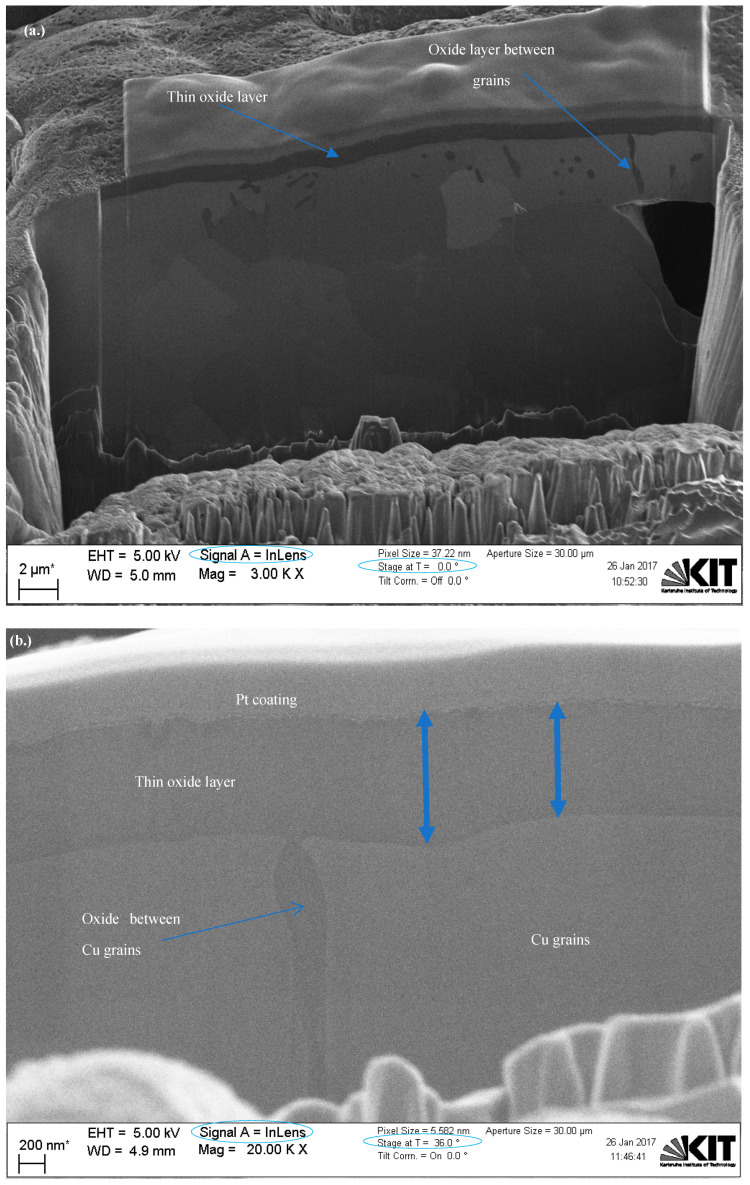
Dual FIB-SEM images of Cu particles with less than 149 µm with a (**a**) 0^°^ and (**b**) 36° angle tilt for thin layer measurement.

**Figure 8:**
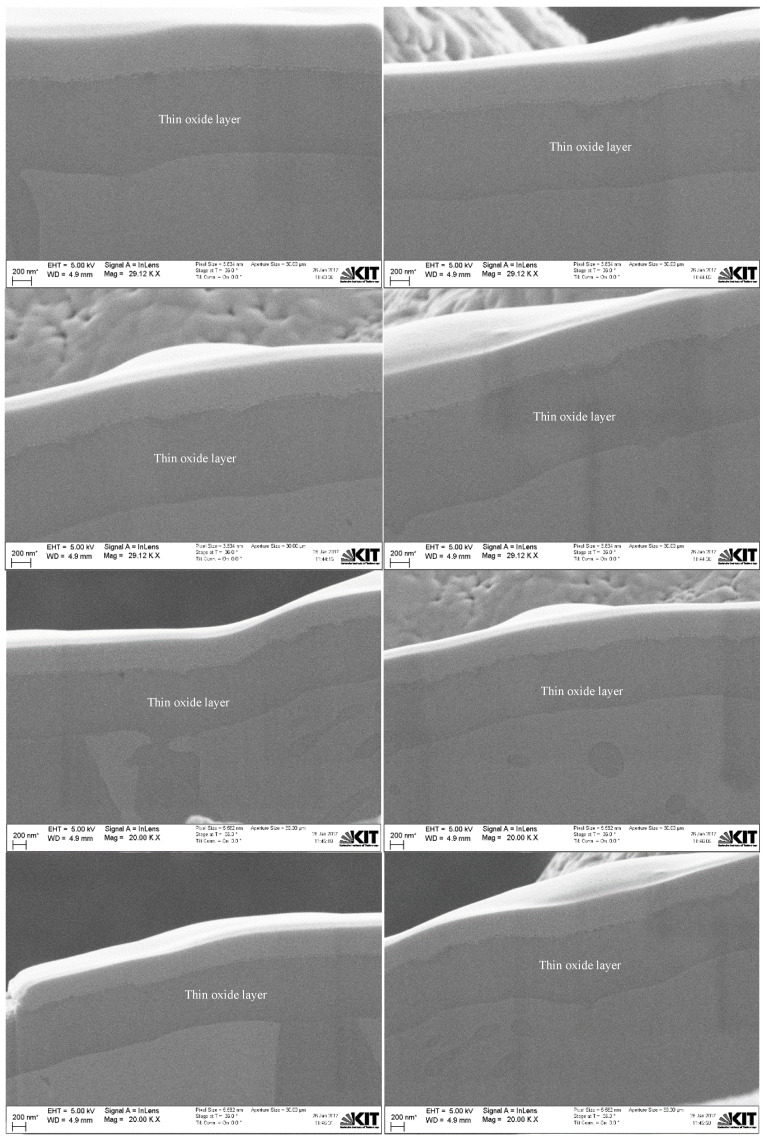
Dual FIB-SEM images of Cu particles with less than 149 µm with InLens detector at 36° stage tilt at various positions.

Figure 6 shows dual FIB-SEM sectioned images for Cu particles with less than 149 µm using backscattered electron (BSE) and secondary electron (SESI) detector modes for comparison, where the oxide layer clearly appears dark (as shown in both images) with either a 36° or 0° stage tilt, respectively. An immersion lens (InLens) detector mode image gives the best resolution for oxide layer measurements, as shown in Figure 7a, where the oxide layer is clearly shown below the Pt coating, as well as within and between the grains as shown in Figure 7 and Figure 8. The reported values of this oxide layer in this study were obtained using the InLens detector mode at a 36° angle, as shown in Figure 7b. FIB-SEM images confirm that the thin oxide layer appears to be not uniformly distributed across any given single particle and with a variable thickness ranging from around 850 nm to 1050 nm as deduced from several measurements at different positions within the same single particle for three (3) particles as shown in Figure 8. The thin oxide layer can be quantitatively characterized with more confidence using the dual beam FIB-SEM system along with the adapted Pt coating setup and using the InLens detector mode for best resolution at a 36° stage angle tilt.

**Figure 9:**
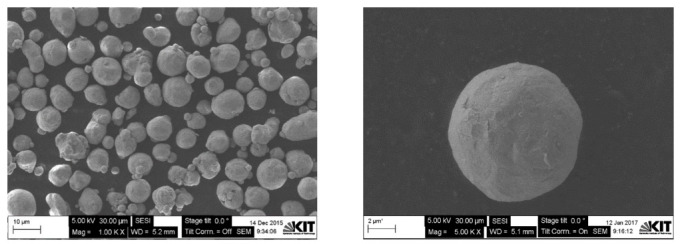
Cu particles with 10 µm APS.

**Figure 10:**
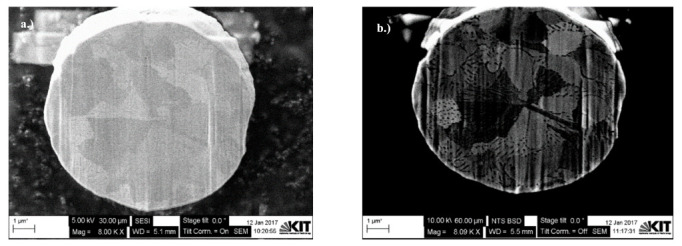
Dual FIB-SEM images of the sectioned Cu particle with 10 µm APS using (**a**) SESI and (**b**) NTS BSD detector modes.

**Figure 11:**
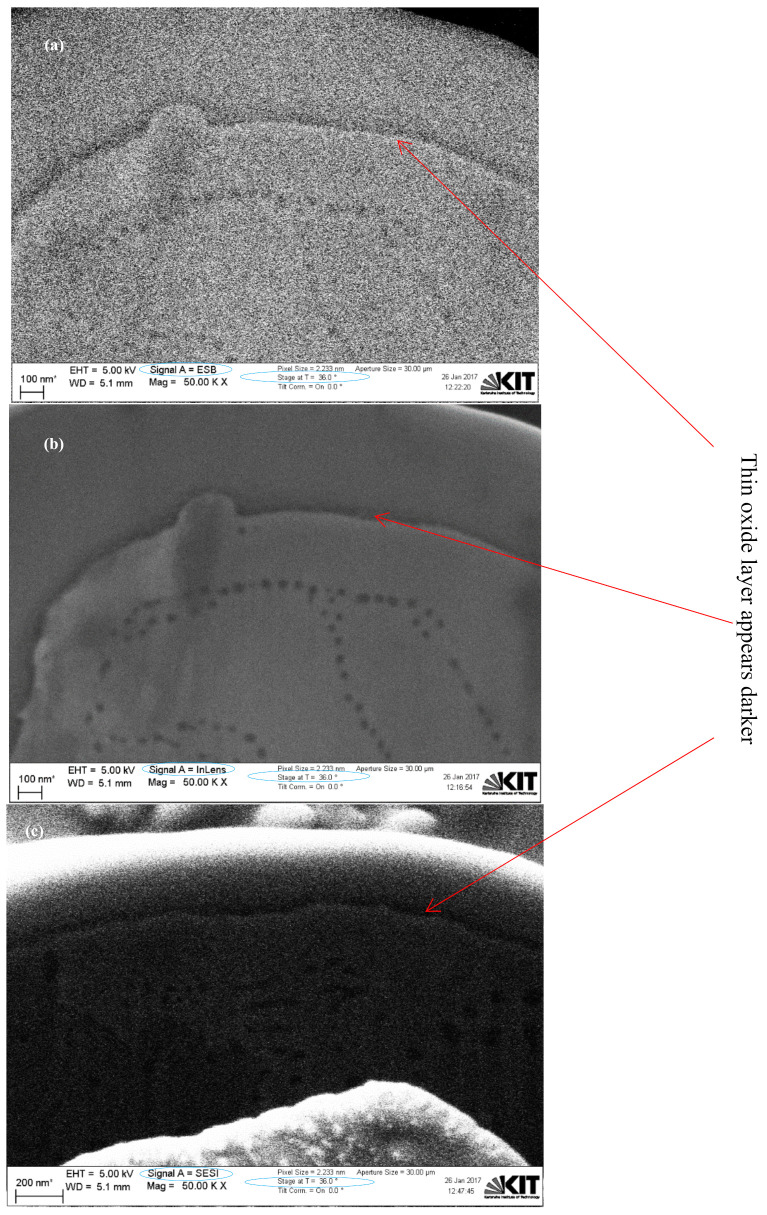
Dual FIB-SEM images for sectioned Cu particles with 10 µm APS using three different detector modes: (**a**) BSE, (**b**) InLens, and (**c**) SESI.

Furthermore, Pt-coated 10 µm APS Cu particles are shown in Figure 9 using the dual FIB-SEM system. Images of the sectioned 10 µm APS Cu particles are shown in Figure 10 using NTS BSD and SESI detector modes at a 0° stage tilt for comparison, where grain boundaries with different orientations, thin oxide layers, and oxides within and between the grains were observed in both modes with more contrast in NTS BSD mode relative to SESI mode. Both detector modes can be very useful to study the grain orientations. Dual FIB-SEM sectioned images of 10 µm APS Cu particles using BSE, InLens, and SESI modes at a 36° stage tilt are shown in Figure 11 for comparison. The thin oxide layer is shown by a dark color in all images; InLens mode again showed the best resolution for thin oxide layer characterization and estimation of a particle’s size as well.

**Figure 12:**
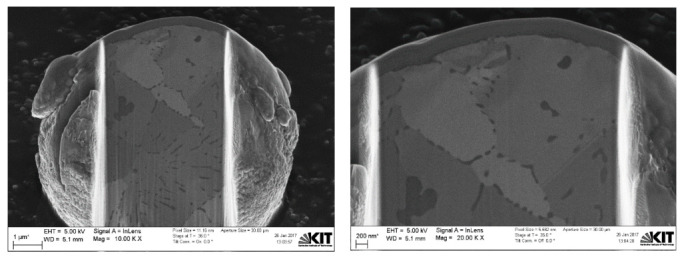
Dual FIB-SEM images with the InLens detector mode of a sectioned 10 µm APS Cu particle.

**Figure 13:**
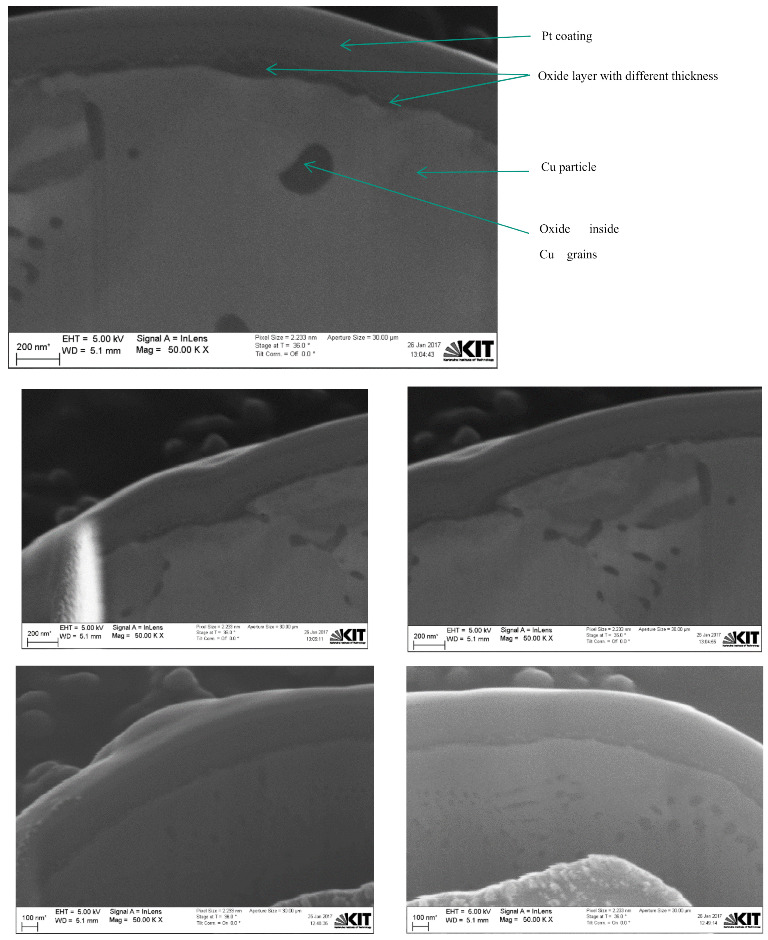
Dual FIB-SEM images of Cu particles with 10 µm APS with the InLens detector at a 36° angle tilt at various positions.

Based on that, the InLens detector mode with a 36° stage tilt was also used to estimate the thin oxide layer on Cu particles with 10 µm APS at different positions, as shown in Figure 12 and Figure 13. The FIB-SEM images indicate that the thin oxide layer on 10 µm APS Cu particles is again not homogenously distributed over a single given particle, with a thickness range from around 22 nm to 67 nm using three particle images. In addition to the thin oxide layer on the surface of the spherical Cu particles, Cu oxide was also detected and observed inside the Cu grains and along the grain boundaries, as shown in both figures. Based on those observations, the oxygen content determined by the CGHE method is mainly related to the thin oxide layer over the particle surface and also related to a minor degree to the oxygen content between the grain boundaries and inside the grains.

## 4. Conclusions

The thermodynamically stable native thin oxide layer “ceramic” formed under normal handling conditions on the different investigated particle sizes of Cu metal powders was qualitatively characterized successfully using AES measurements, with a rough thickness estimation due to AES method limitations and electron beam-induced reduction. XRD pattern characterization established the typical FCC crystal structure of Cu metal powder with a lattice parameter of 3.61500 Å and a space group of Fm-3m with no detection of any kind of Cu oxide peaks due to the detection limits of the XRD technique. On the other hand, quantitative characterization of the oxide layer was successfully achieved using a dual-beam FIB-SEM system and the CGHE method. The oxygen content of the as-received Cu powders was found to have a mean value of around 0.575 wt%, with a detection limit of 0.006 wt% using CGHE. This thin oxide layer was quantitatively characterized with more confidence using the dual beam FIB-SEM system. The Pt-coated Cu particles were sliced via in-situ FIB sectioning at a 0° stage tilt followed by oxide layer thickness characterization using SEM imaging at a 36° stage tilt to avoid any stage tilt correction, with best resolution when using InLens detector mode as compared to the other detector modes used (SESI, BSE and NTS BSD). The thin oxide layer was found to be inhomogeneously covering the particle surface in the two investigated Cu metal powders, where its thickness was strongly dependent on the particle’s sizes. Its thickness was in a range from around 22–67 nm for 10 µm APS Cu particles and from around 850 nm to 1050 nm for the particles with less than 149 µm. Cu oxide was also observed inside the grains and along grain boundaries in minor amounts. This study finding could be useful for a better fundamental understanding of MW–material interaction during metal powder sintering. Moreover, it provides useful input values for numerical models to accurately estimate the effective dielectric and magnetic properties of a given metal powder during MW heating for better control, and will be of wide use for MW processing in metal sintering or other related fields where metal powder particles are being heated under electromagnetic waves.

## Figures and Tables

**Table 1:** Two different spherical-particle Cu metal powder specifications.

Type	Particle Size	wt%
Cu spherical less than 149 µm 99.95% Stock Nr. 11 070	less than 44 µm	53.8%
	greater than 44 µm–less than 74 µm	24.0%
	greater than 74 µm–less than 149 µm	21.9%
	greater than 149 µm	0.3%
Cu spherical APS 10 µm 99.9% Stock Nr. 42 689	less than 7.39 µm	10%
	less than 9.78 µm	50%
	less than 14.05 µm	90%

**Table 2:** Oxygen content measurement (wt%) of the two Cu metal powders.

Detection Limit (wt. %)	Cu Powder with 10 µm APS		Cu Powder with Less Than 149 µm	
	Oxygen Content Mean (wt.%)	SD	Oxygen Content Mean (wt.%)	SD
0.006	0.573	0.012	0.578	0.037

SD: standard deviation.
